# A Novel Flow Cytometric Approach for the Quantification and Quality Control of *Chlamydia trachomatis* Preparations

**DOI:** 10.3390/pathogens10121617

**Published:** 2021-12-12

**Authors:** Romana Klasinc, Michael Reiter, Astrid Digruber, Waltraud Tschulenk, Ingrid Walter, Alexander Kirschner, Andreas Spittler, Hannes Stockinger

**Affiliations:** 1 Institute for Hygiene and Applied Immunology, Center for Pathophysiology, Infectiology and Immunology, Medical University of Vienna, 1090 Vienna, Austria; michael.a.reiter@meduniwien.ac.at (M.R.); alexander.kirschner@meduniwien.ac.at (A.K.); hannes.stockinger@meduniwien.ac.at (H.S.); 2 Institute of Microbiology, Department of Pathobiology, University of Veterinary Medicine Vienna, 1210 Vienna, Austria; Astrid.Digruber@vetmeduni.ac.at; 3 Institute of Morphology, Department of Pathobiology, University of Veterinary Medicine Vienna, 1210 Vienna, Austria; Waltraud.tschulenk@vetmeduni.ac.at (W.T.); Ingrid.walter@vetmeduni.ac.at (I.W.); 4 Division Water Quality & Health, Department Pharmacology, Physiology and Microbiology, Karl Landsteiner University of Health Sciences, 3500 Krems, Austria; 5 Core Facility Flow Cytometry and Department of Surgery, Research Laboratories, Medical University of Vienna, 1090 Vienna, Austria; andreas.spittler@meduniwien.ac.at

**Keywords:** flow cytometry, *Chlamydia trachomatis*, quantification, viability, quality control

## Abstract

*Chlamydia trachomatis* is an obligate intracellular pathogenic bacterium with a biphasic developmental cycle manifesting two distinct morphological forms: infectious elementary bodies (EBs) and replicative intracellular reticulate bodies (RBs). Current standard protocols for quantification of the isolates assess infectious particles by titering inclusion-forming units, using permissive cell lines, and analyzing via immunofluorescence. Enumeration of total particle counts is achieved by counting labeled EBs/RBs using a fluorescence microscope. Both methods are time-consuming with a high risk of observer bias. For a better assessment of *C. trachomatis* preparations, we developed a simple and time-saving flow cytometry-based workflow for quantifying small particles, such as EBs with a size of 300 nm. This included optimization of gain and threshold settings with the addition of a neutral density filter for small-particle discrimination. The nucleic acid dye SYBR^®^ Green I (SGI) was used together with propidium iodide and 5(6)-carboxyfluorescein diacetate to enumerate and discriminate between live and dead bacteria. We found no significant differences between the direct particle count of SGI-stained *C. trachomatis* preparations measured by microscopy or flow cytometry (*p* > 0.05). Furthermore, we completed our results by introducing a cell culture-independent viability assay. Our measurements showed very good reproducibility and comparability to the existing state-of-the-art methods, indicating that the evaluation of *C. trachomatis* preparations by flow cytometry is a fast and reliable method. Thus, our method facilitates an improved assessment of the quality of *C. trachomatis* preparations for downstream applications.

## 1. Introduction

*Chlamydia trachomatis* remains the most commonly reported sexually transmitted bacterial pathogen in the world [1]. The WHO estimated that there were 131 million new cases of chlamydial infection globally among adults in 2012 [2]. In the United States alone, where prevention programs benefit from strong data provided by the Centers for Disease Control and Prevention, nearly two million cases have been reported annually, with an increasing rate since the year 2000 [3]. The infection caused by *C. trachomatis* manifests a broad spectrum of distinct clinical symptoms, which are also conditioned by a strict tissue tropism determined by different serovars. Serovars A to C are responsible for the leading cause of blindness in developing countries, where strategy programs have been initiated to eliminate trachoma [4]. In contrast, serovars D to K represent the most prevalent sexually transmitted organism in high-income countries [5]. Serovars L1 to L3 cause invasive urogenital and anorectal infections with an increasing incidence among HIV-infected men who have sex with men [6].

*C. trachomatis* harbors one of the smallest bacterial genomes. However, this obligate intracellular bacterium presents a manifold biological background with a complex biphasic developmental cycle, which is characterized by two distinct morphological forms: (i) the infectious elementary body (EB); and (ii) the replicative intracellular reticulate body (RB). During the course of an infection, EBs bind to susceptible host cells and enter as phagocytic vesicles. In these vesicles, the developing EBs form the chlamydial inclusion, where each individual EB differentiates into one RB. RBs depend on host cell metabolites to develop and divide by binary fission. After several divisions, RBs differentiate into EBs, which finally leave the host cell by lysis or extrusion [7]. Size is a main morphological trait serving to discriminate between the distinct stages. At 300 nm, chlamydial EBs are considerably smaller than RBs, which are approximately 1000 nm [8]. In addition, the intermediate body (IB), a transitional stage between the distinct EBs and RBs, has been described.

The state-of-the-art method to purify EBs and RBs is density gradient centrifugation followed by the assessment of infectious particles by titration of inclusion-forming units (IFUs) using immunofluorescence of the infected host cells [9]. To determine viability, a comparison of the IFU titer with the total bacterial counts is required [10]. Other options to assess *Chlamydia* viability are viability PCR and messenger RNA detection [11]. However, the limitations of these methods are uncertainties concerning the degradation and stability of messenger RNA, strong dependency on membrane integrity, and bacterial load.

Flow cytometry-based methods to measure viability have been successfully applied to other microorganisms, including bacteria from water samples and cultured bacteria, such as *Escherichia coli*, *Pseudomonas aeruginosa* and *Legionella* species [12,13,14,15,16,17]. In fact, flow cytometric methods have been also performed for analyses of infectivity, persistence, and growth of Chlamydia within eukaryotic host cells [18,19,20]. However, these studies did not determine the quantity and viability of purified Chlamydia preparations. Only one study by Vromman and colleagues performed direct measurement of EBs by flow-cytometry without presenting a detailed methodology or including viability measurements [21]. Although both the number of EBs and their viability affect the outcome of an infection [22,23], respective methods to determine these parameters in *C. trachomatis* preparations are rarely utilized and compared [10,24]. Therefore, we analyzed in this study whether flow-cytometry can be used for quantification and qualification of EBs in purified preparations despite their small size that makes them hardly distinguishable from background noise. We also hypothesized that it might be possible to use viability markers already used in flow-cytometry for an objective evaluation of the viability of *C. trachomatis* preparations. Finally, we tested whether we are able to distinguish different developmental stages of *C. trachomatis* by flow-cytometry and how the new methodology can be brought into connection with already well-established methods. In our opinion, a precise quality control of *Chlamydia* preparations is a prerequisite when performing, in particular, infectivity assays or experiments with immune cells in which it is necessary to discriminate between viable/infective and non-viable stages. To the best of our knowledge, a detailed methodology for a host cell-independent quantitative assessment of chlamydial infectious particles has not yet been established. Therefore, we develop and describe here a novel flow-cytometric method for the simple and time-saving quantification and viability assessment of *C. trachomatis* preparations.

## 2. Results

### 2.1. TEM of C. trachomatis Preparations

**Figure 1:**
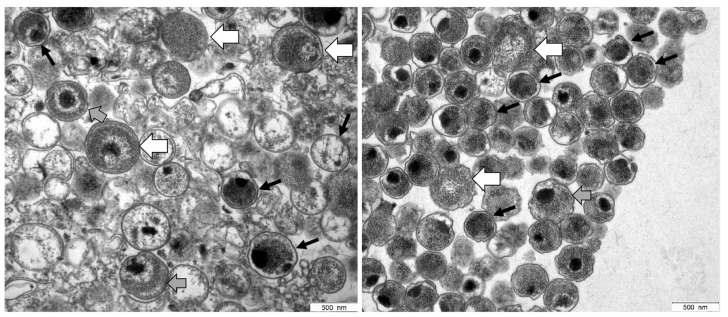
Electron micrographs of *C. trachomatis* serovar E (left panel) and serovar L2 (right panel) samples prepared by using Gastrografin^®^ gradient centrifugation. Based on their characteristic morphological features, a mixture of EBs and RBs was detected in both preparations. EBs (black arrows) contain highly condensed material appearing as electron-dense and electron-lucent, whereas RBs (white arrows) represent larger structures containing relaxed, reticulated nucleoid material. Intermediate morphologies where characteristics of both are observed may represent IBs (grey arrows).

The ultrastructural analysis of preparations showed the presence of EBs characterized by small size (∼300 nm diameter) and highly condensed chromatin, as well as larger RBs (up to 1000 nm diameter) with relaxed chromatin (Figure 1). Because the EB-enriched fraction collected at the 44/54% interface also contained RBs, it is thus referred to in the following as EB/RB preparation.

### 2.2. Measurements of DPCs, Sample Characteristics and Voltage Optimization

**Figure 2:**
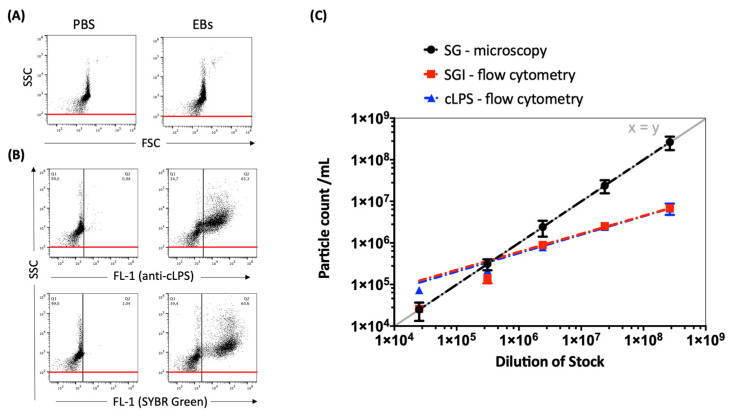
(**A**) Representative dot plots of *C. trachomatis* (serovar E) EB/RB preparations, side scatter (SSC) vs. forward scatter (FSC), compared to phosphate-buffered saline (PBS). (**B**) Representative dot plots of *C. trachomatis* preparations (concentration of 2.39 × 10^6^/mL) stained with either FITC-conjugated cLPS mAb B410F or nucleic acid stain SGI; SSC (488-10+OD2 filter) vs. fluorescence intensity (FL-1, emission filter 530/30 nm, log scale) is shown. PBS supplemented with both dyes without EBs/RBs was measured to determine the background noise; fluorescence cut-off values were set at 1% (black line) and the SSC threshold at 100 (red line). (**C**) Counts of SGI- vs. cLPS-stained EB/RB-dilutions (from 1:100 to 1:1,000,000) measured by flow cytometry compared to the DPCs of SYBR^TM^ Gold (SG)-stained EBs/RBs analyzed by epifluorescence microscopy. The exponential trend lines were fitted and shown with a graph of standard linear relation (y = x, grey line).

Preparations of *C. trachomatis* were stained with either cLPS mAb or nucleic acid stain SYBR^®^ Green I (SGI). Comparative measurements show no significant differences in particle counts between the staining methods when using flow cytometry. These results indicate that SGI nucleic acid stain is a convenient alternative for the direct enumeration of *C. trachomatis* stock preparations with a no-wash-out staining protocol to enable the quantification of preparations without loss of material. Therefore, SGI was used in the following experiments and for setting optimization. However, significant differences in particle counts are seen between direct particle counts (DPC)-microscopy and DPC-flow-cytometry at serial dilutions from 1:100 to 1:10,000 (Figure 2). Therefore, to improve the comparability between microscopy and flow cytometry, we performed further adaptations in terms of optimized voltage and threshold settings.

### 2.3. Impact of Thresholding and Sample Dilution on Event Counts and Coincidence

**Figure 3:**
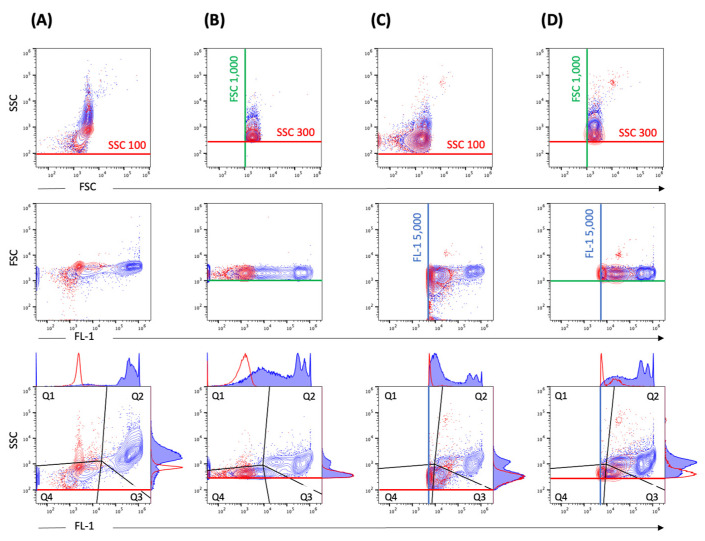
Standard instrument filter settings. Representative contour plots of EBs/RBs (serovar E) at a concentration of 2.39 × 10^7^/mL stained with SGI (blue) in comparison to SGI-incubated PBS reagent control (red). The measurement was performed with different threshold settings: (**A**) SCC 100, (**B**) SSC 300 and FSC 1000, (**C**) SCC 100 and FL-1 5000, (**D**) SSC 300 and FSC 1000 and FL-1 5000. The thresholds are marked with corresponding lines (SSC 100 or SSC 300—red, FSC 1000—green, FL-1 5000—blue). The gating strategy (black lines) including the corresponding histograms with a definition of the positive population in gate quadrant 2 (Q2) is shown in the last row.

**Figure 4:**
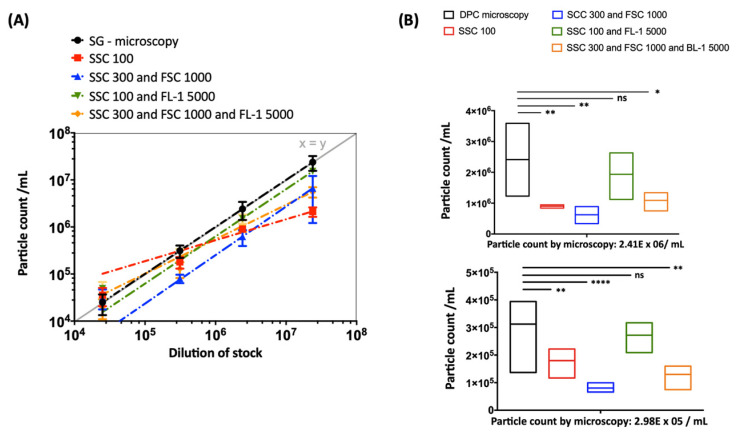
Strong dependency of the particle count on the threshold settings when using standard instrument filters. (**A**) Serial dilutions (from 1:1,000 to 1:1,000,000) of EBs/RBs (serovar E) counted by fluorescence microscopy (DPC microscopy) compared to measurements by flow cytometry (Figure 3, gate Q2) at different thresholds (log scale) with fitting exponential trend lines. (**B**) The statistical analysis was done using one-way ANOVA and Dunnett’s multiple comparisons test. *p* values < 0.05 were considered statistically significant; * *p* < 0.05, ** *p* < 0.01, **** *p* < 0.0001. The analysis is exemplified at two dilutions: 2.41 × 10^6^/mL (upper panel) and 2.98 × 10^5^/mL (lower panel).

Comparative measurements of different threshold settings were performed at different serial dilutions. Different threshold settings show specific contour plot profiles (Figure 3). The DPCs measured by flow cytometry—when including the fluorescence (FL-1 5000) and SSC (SSC 100) threshold (Figure 3C)—were comparable to DPC by microscopy (*p* > 0.05). Other threshold settings did not achieve the same accuracy (Figure 4). In addition, we observed alterations in height (SSC-H) vs. area (SSC-A) plots between measurements with different threshold settings (Figure S1). The principle of height and area is described below in Materials and Methods.

### 2.4. Measurements Performed Using the Small Particle Filter and Identification of Populations

**Figure 5:**
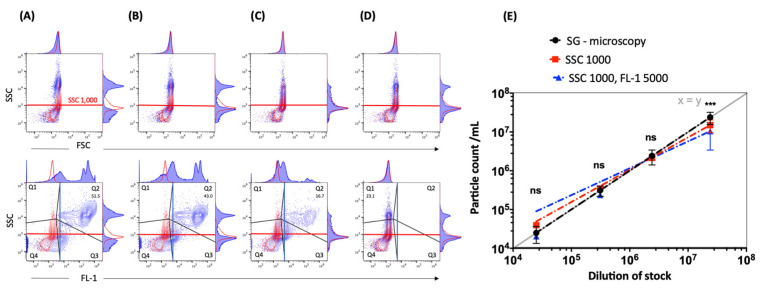
Superior discrimination of EBs/RBs with implementation of a small particle SSC filter. Representative contour plots with corresponding histograms of SSC (SSC 488-10 filter) vs. FSC or FL-1 fluorescence intensity (log scale, FL-1: 530/30-nm filter) of EBs/RBs (serovar E) at the following concentrations: (**A**) 2.39 × 10^7^/mL, (**B**) 2.41 × 10^6^/mL and (**C**) 2.98 × 10^5^/mL stained with SGI. The positive population is shown in gate Q2. PBS stained with SGI (red) is used as a reagent control. (**D**) Unstained EBs/RBs (blue) at 2.41 × 10^6^/mL are shown in gate Q1 and compared to PBS (red). For presentation purposes the threshold was set on SSC 100 and completed with threshold marks used in further measurements: SSC 1000 (red lines), FL-1 5000 (blue lines). The numbers represent percentages of EB/RB preparations (blue contour plots) in the positive gate. (**E**) Measurements of particle counts with new threshold settings. Serial dilutions (from 2.39 × 10^7^/mL to 2.5 × 10^4^/mL) of EBs/RBs (serovar E) counted by fluorescence microscopy (DPC microscopy, black trend line) compared to measurements by flow cytometry (gate Q2) without (SSC 1000) or with (SSC 1000 + FL-1 5000) a fluorescence trigger. The exponential trend lines were fitted with corresponding equations and R-squared values. The statistical analysis was done using one-way ANOVA and Dunn’s multiple comparisons test. *p* values < 0.05 were considered statistically significant; *** *p* < 0.001.

Consecutive measurements of the samples were performed by implementing the SSC 488-10 filter. A good separation of populations in the SSC was possible at different dilutions of the cell suspension (Figure 5A–C). This approach even made it possible to enumerate unstained particles at a concentration of 1 × 10^6^–5 × 10^6^ particles/mL (Figure 5D). This underscores the importance of appropriate dilution. The filter allows the omission of the fluorescence trigger without compromising the coincidence rate and, consequently, comparable particle counts using only the SSC trigger were achieved (Figure 5E). The advantage of measuring without the fluorescence trigger is the possibility to also visualize the unstained sample at the recommended coincidence rate.

### 2.5. Measurements of Viability by Flow Cytometry

**Figure 6:**
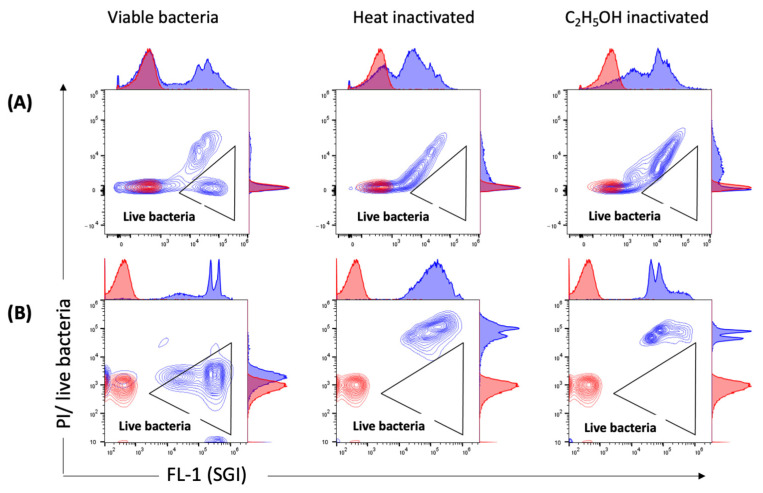
Measurements of membrane integrity by SGI/PI staining. Representative contour plots of viable, heat- and C_2_H_5_OH-inactivated (**A**) *C. trachomatis* EBs/RBs (serovar E) and (**B**) *E. coli* stained with SGI/PI are shown in blue. PBS stained with SGI/PI as a reagent control is shown in red. PI-negative (live) bacteria with intact outer membranes are detected in the triangular gate. Threshold settings: SSC 1000.

**Figure 7:**
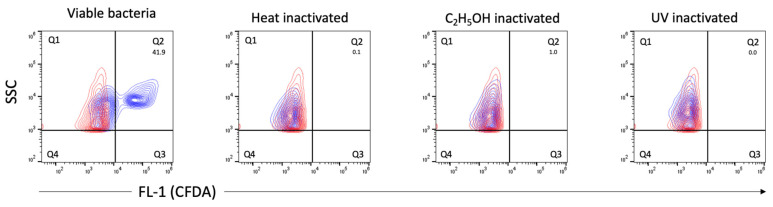
Measurements of esterase activity by CFDA staining. Representative contour plots of viable, heat-, C_2_H_5_OH- and UV-inactivated *C. trachomatis* (serovar E) EB/RB preparations. CFDA-positive, esterase-producing bacteria are shown in blue and detected in gate quadrant 2 (Q2), the numbers represent percentages of viable bacteria. PBS stained with CFDA as a reagent control is shown in red. Threshold settings: SSC 1000.

We also applied flow cytometry to employ viability indicators (such as membrane integrity and esterase activity, previously used with numerous other bacterial species) for the analysis of viable bacteria in *C. trachomatis* EB/RB preparations including serovars E, F and L2. The EB/RB preparations did show considerable amounts of propidium iodide (PI)-positive particles (from 48.2 to 73%) in fresh chlamydial stocks, indicating that the majority of bacteria had compromised cell wall integrity. This consideration is supported, first, by comparing the fresh EB/RB preparation after its inactivation with heat or ethanol; in the latter, the PI-negative population shifted towards positivity (Figure 6A). Second, viable *E. coli* preparations did not score PI-positively unless treated with heat or ethanol, as shown in Figure 6B. Third, the 5(6)-carboxyfluorescein diacetate (CFDA) staining of viable *C. trachomatis* preparations showed positive particles accounting from 39.3 to 60.5%. The inactivated preparations did not show any remaining esterase activity (Figure 7).

**Figure 8:**
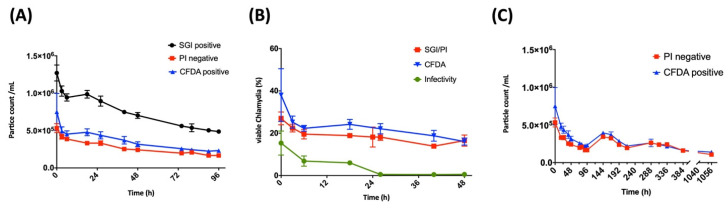
Viability of *C. trachomatis* measured by flow cytometry over time. (**A**) Time course of SGI/PI- and CFDA-stained EBs/RBs counts at the same dilution over 0 to 96 h. (**B**) Measurements of membrane integrity or metabolic activity of EBs/RBs (serovar E, Stock 1CTE) measured by flow cytometry compared to infectivity. The infectivity is calculated from the quotient between IFUs and the DPC measured by epifluorescence microscopy. (**C**) Long-term measurements of PI-negative and CFDA-positive particle counts during 0 to 44 days (1056 h).

Moreover, by flow cytometry, we also analyzed *C. trachomatis* samples over a time course of 96 h and compared the particle counts per mL (Figure 8A). There was a significant decrease in the number of particles over this time from 1.27 × 10^6^/mL to 4.86 × 10^5^/mL, probably due to decay of EBs or aggregation of single particles. This decline must be considered when calculating percentages of metabolically active particles as fractions of DPCs. The viability stains analyzed over a time course of 48 h showed the strongest decrease within the first six hours, from 38.0% to 22.3% and from 27.0% to 19.6% for CFDA and SGI/PI, respectively (Figure 8B). In addition, esterase activity and membrane integrity measured by flow cytometry were aligned with infectivity, which is defined as the ratio between IFUs and the DPCs measured by epifluorescence microscopy. The infectivity of freshly thawed stocks was 15.4% at baseline and already decreased to below 1% after 24 h. This was in contrast to both viability stains measured by flow cytometry, which remained stable after six hours and did not fall below 16% within 48 h (Figure 8B).

Generally, the viability stains by flow cytometry showed higher percentages of viable bacteria in comparison to infectivity, but not to the same extent when comparing different serovars (see below). However, at a later time point—especially after 24 h—the discrepancy between infectivity and viability by flow cytometry became evident. Strikingly, the metabolic activity and membrane integrity of the *Chlamydia* species remained intact even after 44 days in a host-free environment at 4 °C and did not drop below 1 × 10^5^/mL (Figure 8C).

### 2.6. Measurements of DPCs and Viability by Flow Cytometry in Comparison to Other Methods

To evaluate different preparations (including different serovars) of *C. trachomatis*, the freshly thawed stocks were diluted and compared with the results either gathered by standard methods or by our new methods using flow cytometry. The summary of these comparative measurements is shown in Table 1, indicating very good comparability and reproducibility of the DPCs measured by microscopy or flow cytometry for all serovars. Comparing the viability methods, the differences depended on the given serovar. Serovar L2 showed the highest CFDA activity (50.1%) compared to infectivity (10.5%) and membrane integrity (37.8%), whereas for serovar E, the differences were less distinct. Serovar F showed a high esterase activity (49.7%) as well as membrane integrity (51.8%), despite relatively low infectivity (8.7%).

## 3. Discussion

Measuring small particles (<500 nm) by flow cytometry is challenging due to the limited resolution and sensitivity of most flow cytometers. Therefore, each approach requires custom-tailored methods, and special adaptations of the flow cytometry settings must be taken into account. These adaptations include threshold settings on a fluorescence trigger or even combinations of different threshold parameters, while considering that the usual background discrimination in the FSC and SSC is often not feasible. Guidelines for measuring small particles have been established to a large extent by investigating extracellular vesicles (EVs), for which standardization procedures have been published [25,26,27]. Apart from EV research, another rapidly growing field for flow cytometry applications is the analysis of bacterial or archaeal cells in natural environments. In particular, fastidious bacterial species which are difficult to cultivate are very eligible for flow cytometry-based quantification [28].

This includes *C. trachomatis*, which is difficult to cultivate due to its intracellular growth and for which the methods of quantification are conspicuously time-consuming. Moreover, to avoid observer bias and facilitate reproducible results, strictly standardized procedures are needed. Recent advances in flow cytometry have paved the way for new applications and thereby represent a means to overcome these limitations. Especially high-resolution or nanoscale flow cytometry enables single-cell discrimination of submicron particles under certain conditions. However, when studying small particles, the following adjustments have to be considered: First, it must be noted that there are significant differences between flow cytometers and most of them do not have nanoparticle sensitivity, which is the prerequisite to detect particles smaller than 500 nm [29]. Provided that the available flow cytometer enables small particle discrimination, the next step is to identify the positive population by choosing an appropriate staining method. For bacteria, nucleic acid dyes of the SYBR^®^ Green family have been successfully used for DPCs [13,30]. This approach was later extended even to free-living viruses [31,32]. We utilized this technique and applied it in a new manner to enumerate chlamydial stocks. No differences in terms of particle counts were detected when comparing the DPCs of samples stained with anti-cLPS-specific mAb or with SGI. Nevertheless, the SGI-stained samples provided a brighter signal and facilitated a very simple staining technique (Figure 2).

To establish a user-independent flow cytometry-based counting method for *Chlamydia*, we then estimated the appropriate dilution of the samples. Furthermore, it was essential to set the right threshold for separating populations of interest from the background noise. Both parameters strongly influenced the number of measured events per second and consequently also the coincidence rate or swarming (Figure S1). Coincidence describes the simultaneous passing of two or more particles through the interrogation point of the laser, while still being distinguishable [33,34]. In contrast, swarming occurs in highly concentrated samples when the coincidence progresses to a permanent scatter and fluorescent signal and it is no longer possible to separate individual events [35,36]. Allowance must be made for these two phenomena when measuring small particles and focusing on the enumeration of total counts. To prevent the occurrence of either phenomenon, it was necessary to use serial dilutions of chlamydial preparations after adapting the flow cytometer settings. Our results showed an event rate displaying a linear correlation of the SGI-positive particle concentration upon serial dilution, indicating a good separation of single events. However, despite linearity, the DPC measured by flow cytometry was only comparable to the DPC by microscopy at lower dilutions and showed strong dependency on the threshold settings (Figure 2). This may be explained by the influence of non-fluorescent submicron-sized particles on the light scatter detection of fluorescent-labeled events of interest, as previously described by Libregts and colleagues [37].

*C. trachomatis* preparations purified by using density gradient centrifugation potentially contain particles representing membrane vesicles from the host cell. Nevertheless, when using the right flow cytometer settings under consideration of the appropriate serial dilution, the DPC by flow cytometry showed high reproducibility (Figure 4 and Table 1). After including the small particle filter, an increase in the dynamic range of the SSC detection was achieved. Consequently, it was possible to separate chlamydial EB/RB preparations from background noise by applying only the SSC trigger. Therefore, including the fluorescence trigger was no longer a prerequisite for accurate DPC (Figure 5). This approach with the visualization of the unstained control is closer to the analysis of, e.g., human cells and may be easier for the user to comprehend. It thus may also represent a more convenient method to incorporate additional markers and perform a multi-panel stain including compensation more approachable in a “small particle setting”. This specific small particle filter also proved to be very valuable for the flow cytometry-based methods of viability detection. To assess the viability of *C. trachomatis* preparations, we modified previously described viability methods of bacteria [12,13,14,15,16,17]; a summary is shown in Figure S2. As an indicator of esterase activity, the fluorogenic substrate CFDA was previously used as a marker of bacterial viability [12,13]. The principle of action is the passive entrance of the substrate into the cell, followed by esterase-mediated hydrolyzation. This leads to the generation of a highly fluorescent fluorophore when an intact cell membrane protects the esterase from degradation [12,38].

We applied another method to access membrane integrity by adding PI to SGI-stained samples (Figure 7). PI is a nucleic acid-staining dye that, unlike SGI, only enters cells with damaged membranes. It has been widely used previously to identify viable bacteria in water samples [13,39,40] and bacterial cultures [41]. With both staining methods, we identified viable *C. trachomatis* in our preparations. However, when comparing the results with the SGI/PI staining of *E. coli* (Figure 6B), we clearly demonstrated that even fresh preparations of chlamydial stocks showed high percentages of PI-positive particles (Figure 6A). This finding indicates a high proportion of EB/RBs with damaged membranes, addressing once more the issue of dead EBs in even highly purified preparations [42,43].

This observation contrasts with the findings of Vromman and colleagues, where the DPC of purified green fluorescent protein (GFP) expressing *Chlamydia* measured by flow cytometry was equal to the IFU [24]. One could explain this difference by a weak signal of GFP in certain EBs that consequently results in an underestimation of measured particles.

The calculated infectivity of our samples was generally lower in comparison to the flow cytometry-based viability methods. This may be explained by the fact that not all viable cells are infective, but also viable cells can be overestimated in a preparation. However, although viability cannot be equated with infectivity, through the correlation given between measurements of particle counts by DNA staining, the viability staining and the infectivity (IFU), we can extrapolate which proportions of infectious versus non-infectious or dead *Chlamydia* are present in our preparations. Strikingly, there were considerable differences when analyzing different serovars. Serovar F, in particular, showed the highest percentages of viable cells by both flow cytometry-based methods, despite the lower level of infectivity (Table 1). Serovars E and L2 showed a pronounced difference between CFDA-positive and PI-negative cells. These differences diminished over time, as shown by the long-term viability measurement of serovar E (Figure 8C). Currently, we cannot explain the reason for the pronounced difference in esterase activity in serovars E and L2, also when compared to the lower proportion of *C. trachomatis* with intact membranes. A prolonged but also less specific persistence of esterase activity in certain samples may be the reason for this difference. This issue was briefly addressed in a study [38] in which esterase activity was measured in gamma-radiated cells for over two weeks. Another possibility may also be the presence of host cell-derived CFDA-positive components in chlamydial preparations that contribute to the elevated levels. However, when performing the same viability assays with inactivated EB/RB preparations (ethanol-, heat- or UV-treated samples), both staining methods stood out as highly reproducible and showed neither residual metabolic activity nor membrane integrity (Figure 6 and Figure 7). Both independent viability parameters CFDA- and PI/SGI staining significantly correlate even after long-term measurements of stored *Chlamydia* samples in a host-free environment, and further correlate with the IFU (Figure 8B,C). Finally, both dyes were associated with long-term stability, indicating that a respectable proportion of cells still exhibited viability markers after storage in a host-free environment at 4 °C (Figure 8C).

Early research in chlamydial metabolism regarded EBs as metabolically inert particles [44,45]. More recently, evidence has emerged that offers contradictory findings and requires this hypothesis to be reconsidered, since an increasing number of studies have demonstrated metabolic activity involving transcription and protein biosynthesis in a host-free environment in chlamydial EBs [10,46,47]. This is also in accordance with our results that show long-term (44-day) esterase activity as well as membrane integrity of serovar E measured by flow cytometry (Figure 8C). However, the importance of the prolonged metabolic activity during host-free incubation of *C. trachomatis* despite the rapid decrease in infectivity needs further clarification.

SGI staining made it possible to gain specific flow cytometry profiles with a differentiation of two populations in the fluorescence channel (Figure S3A). These two fractions may represent the different developmental stages of *C. trachomatis*—EBs or RBs—with a high or low content of nucleic acids, or may reflect differences in the ratio between double-stranded DNA and RNA. Intriguingly, in serovar L2, this differentiation was even seen by PI staining, in which the population with lower FL-1 signal intensity (P2) was predominantly PI-positive, as we demonstrated by back-gating (Figure S3B). When looking more closely at the PI negative populations in long-term measurements upon storage in a host-free environment, the population in the P2 gate displayed up to 6 h, as two sub-populations and the sub-population with the lowest fluorescence intensity slowly faded away between 6 and 48 h. Furthermore, the percentage of the lower (P2) versus the higher (P1) SGI-stained events shifted over time—74%/22% versus 50%/43% (Figure S4). These less stable sub-populations in gate P2 may represent RBs and/or IBs, suggesting differences in membrane stability between different developmental stages. This is in accordance with the findings of Sixt and colleagues who, using the redox dye 5-cyano-2,3-ditolyl tetrazolium chloride, demonstrated that only EBs and not RBs of *C. aemoebophila* contributed to the metabolic activity after 40 h of host-free incubation [46]. A similar flow cytometry-based discrimination of bacterial populations in natural waters by SGI signal intensity with a high or low content of nucleic acids has previously been discussed [13,48]. However, whether EBs and IBs or RBs in our samples resemble populations with high and/or low nucleic acid staining remains subject for further investigations.

The method presented in our study can also be seen as the basis for the separation and flow-cytometric sorting of subpopulations in *Chlamydia* preparations. For this approach, as for the analysis shown here, appropriate sample dilution and optimized threshold settings are an absolute requirement for coincidence or swarm control. The flow cytometric analysis of *C. trachomatis* preparations including sorting have a great potential, as they facilitate the distinction and separation of subpopulations based on nucleic acid content, viability or specific markers in these preparations. In addition, the identification of appropriate subpopulation markers would help to establish a greater degree of accuracy on this matter. Thus, high-resolution flow cytometry-based analysis can assist in characterizing cell culture-derived bacterial preparations with the potential to identify, apart from the well-known EBs or RBs, membrane vesicles and other, so far unidentified submicron particles. Unfortunately, due to current technical limitations of flow cytometers to sort small particles, we were not able yet to sort the two populations seen in Figures S4 and S5 and prove whether the one or the other represents EBs or RBs, respectively. Finally, our approach is only applicable for purified *Chlamydia* preparations. However, due to contamination with fragments from the host cells, crude preparations are of limited value in experimental settings, in particular when studying host-pathogen interactions and immune responses using in vitro and in vivo immunoassays. Therefore, due to this constraint we do not recommend the use of crude *Chlamydia* preparations but rather purified ones and to analyze their quality by the flow-cytometry based approach described here.

## 4. Conclusions

In conclusion, we developed a highly reproducible flow cytometry-based method to both quantify chlamydial preparations and assess viability. We optimized the flow cytometry settings by implementing a neutral density filter. Thereby, we established a method for a better quantitative and qualitative assessment of *C. trachomatis* preparations for further downstream applications.

## 5. Material and Methods

### 5.1. Microbial Strains and Purification of C. trachomatis Preparations

*C. trachomatis* serovars E (DSM 19131), F (DSM 19410) and L2 (DSM 19102) were used in this study and propagated in HeLa epithelial cells (ATCC^®^ CCL-2^TM^) or McCoy [McCoyB] fibroblasts (ATCC^®^ CRL-1696TM) with modifications, as previously described [9]. The eukaryotic cell lines were regularly tested for mycoplasma contamination by fluorescence microscopy using Hoechst stain No. 33342. In brief, confluent monolayers were infected with *C. trachomatis* and grown in Iscove’s Modified Dulbecco’s Medium with L-glutamine (GibcoTM, Thermo Fisher Scientific Waltham, MA, USA) containing 10 vol% heat-inactivated fetal bovine serum (Biowest, Nuaillé, France; Cat. No. S181H-500). To cultivate serovars E and F, 1 vol% minimum essential medium non-essential amino acids solution (MEM NEAA, 100X, Gibco™, Thermo Fisher Scientific, Cat. No. 11140050) and 1 μg/mL cycloheximide (Sigma-Aldrich, Burlington, MA, USA; Cat. No. 01810) were added to the medium described above. After 48 h post-infection at 37 °C in a 5% CO_2_ atmosphere, the cells were harvested by disrupting the monolayer with a cell scraper. The cell suspension was sonicated using a sonic dismembrator (Model 120, Fisherbrand^TM^) at an amplitude of 30% for 2 × 20 s. The chlamydial EBs/RBs were obtained by sequential centrifugation of the lysates at 500× *g* (15 min, 4 °C) and 27,000× *g* (35 min; 4 °C). The pelleted material was suspended in sucrose-phosphate-glutamic acid (SPG) buffer (0.2 M sucrose, 3.8 mM KH_2_PO_4_, 7.2 mM Na_2_HPO_4_, 5 mM L-glutamic acid, pH 7.4) for further density gradient purification, as previously described [49]. In brief, the partially pure EB/RB preparations were layered onto 20% Gastrografin^®^ (Bayer, Leverkusen, Germany; Cat. No. 80375310) in K-36 buffer (0.1 M KCl, 0.015 M NaCl, and 0.05 M potassium phosphate buffer, pH 7.0) and centrifuged at 40,000× *g* for 30 min in a Beckman SW28 rotor at 4 °C. Further purification followed by overlaying the chlamydial EBs/RBs onto a discontinuous gradient of 34%, 44% and 54% Gastrografin^®^ in K-36 buffer. The EB-enriched fraction was collected at the 44/54% interface after centrifugation at 40,000× *g* for 1 h in a Beckman SW28 rotor at 4 °C. The collected fractions were diluted in 10 times the volume of SPG buffer and centrifuged at 18,650× *g* for 30 min at 4 °C. The pellet was resuspended in SPG buffer and stored in aliquots at −80 °C.

### 5.2. Analysis of C. trachomatis Stocks by Transmission Electron Microscopy

For evaluation and quality control, samples of *C. trachomatis* serovars E and L2 were analyzed by transmission electron microscopy (TEM). This method is broadly used to classify ultrastructural characteristics and distinguish between different morphological forms [10,46]. For this purpose, pellets of density gradient-purified EB-enriched fractions of *C. trachomatis* preparations were fixed in 3% glutaraldehyde (Merck) in 0.1 M Sørensen phosphate buffer (pH 7.4). After washing in Sørensen phosphate buffer, the specimens were postfixed in a 1% solution of osmium tetroxide. Dehydration was performed by a series of graded ethanol solutions (70%, 80%, 96% and 100%) subsequently infiltrated with propylene oxide, followed by increasing ratios of epoxy resin-propylene oxide (1:1, 3:1) and, finally, pure resin. After an additional change, the resin was polymerized at 60 °C. Semi-thin sections were cut at 0.8 μm and stained with toluidine blue, ultra-thin sections were cut at 70 nm, mounted on copper grids (Science Services, Munich, Germany), and stained with uranyl acetate and lead citrate. Transmission electron micrographs were made with an electron microscope (EM900, Zeiss, Oberkochen, Germany).

### 5.3. Titration of C. trachomatis Stocks and Total Cell Counts by Microscopy

Serial dilutions of *C. trachomatis* stocks in SPG were transferred to monolayer tissue culture cells seeded in 24-well plates and centrifuged for 60 min at 900× *g* (room temperature) in a swinging bucket rotor for microtiter plates. After addition of 1 mL chlamydial culture medium (described above), the samples were incubated for 30 to 48 h depending on the specific serovar. After staining with fluorescein isothiocyanate- (FITC) conjugated anti-*Chlamydia* lipopolysaccharide (cLPS) monoclonal antibody (mAb) B410F (Thermo Fisher Scientific, Cat. No. MA1-7339), the number of IFUs was counted using an inverted epifluorescence microscope and calculated, as previously described [9].

In brief, IFUs were counted manually, and selected images of the infected cell cultures were further analyzed by ImageJ to objectify the size of the inclusions. All inclusions with a size from 80 µm^2^ to 700 µm^2^ were considered. The average size of inclusions was 128.4 µm^2^ and 301.1 µm^2^ after 40 h and 60 h of incubation, respectively.

DPC-microscopy of *C. trachomatis* preparations (using a standard epifluorescence microscope) were determined with few modifications, as previously described [10,20,50]. SG nucleic acid stain (Invitrogen, Carlsbad, CA, USA, Cat. No. 10358492) was used instead of acridine orange and samples were filtered through a Whatman^®^ Anodisc inorganic filter membrane (Sigma Aldrich, Merck KGaA, Darmstadt, Germany) with a pore size of 0.2 µm. The filter was mounted on 30 µL of a 1:400 dilution of the SG stock solution and processed further according to Riepl and colleagues [50]. The numbers of bacteria per mL were calculated as an average count of at least 15 randomly chosen grids within a microscopic field. The measurements were repeated at least four times.

### 5.4. Staining of Samples, Total Cell Counts and Viability Assays of C. trachomatis Preparations by Flow Cytometry

The preparations, including different serovars of *C. trachomatis* stocks (previously enumerated by epifluorescence microscopy), were diluted in sterile-filtered (using a 0.22 µm syringe filter) PBS solution (pH 7.4) from 1:100 to 1:1,000,000, accounting for particle counts from 1 × 10^8^–5 × 10^8^ to 1 × 10^4^–5 × 10^4^ particles/mL. For long-time measurements, diluted samples were stored in sterile-filtered PBS at 4 °C at a concentration of 1 × 10^7^–5 × 10^7^ particles/mL for up to 44 days.

Serial dilutions of *C. trachomatis* stocks were stained with cLPS mAb and compared to DPCs after staining with SGI nucleic acid stain (Sigma-Aldrich, Cat. No. S9430). In addition, previously described cultivation-independent assessment methods were applied to measure microbial viability. This procedure included a two-color-based assay containing SGI with PI (Sigma Aldrich, Cat. No. P4170) added as a marker of membrane integrity [15] and a single-color-based assay using CFDA (Sigma-Aldrich, Cat. C8166-25MG) as an indicator of esterase activity [13]. The final concentrations were 3 µM and 25 µM for PI and CFDA, respectively. To compare the results derived from the *C. trachomatis* preparations with other bacteria, *E. coli* (NCTC 9001) suspensions were stained and measured under the same conditions. Inactivated preparations of bacteria were prepared as follows: (i) heat inactivation was performed at 80 °C for 15 min, (ii) for the ethanol-inactivation, 10 µL of stock preparations were diluted in 90 µL sterile filtered 70% ethanol suspensions, and for (iii) the UV-inactivation, dilutions of purified EB-enriched fractions were placed under a UV-C lamp (254 nm; 15 W) at 5 cm distance for 30 min. The measurements of particle counts (DPC-flow-cytometry) using stock aliquots at different serial dilutions were performed by flow cytometry using optimized configuration settings, as described below.

### 5.5. Instrumentation and Flow Cytometer Settings

High-resolution flow cytometry of *C. trachomatis* preparations was performed on an Attune NxT flow cytometer (Thermo Fisher Scientific) equipped with a 488-nm flat-top laser at 50 mW. The standard daily quality control startup procedure was performed as recommended by the manufacturer. The FSC and SSC light was collected from the 488 nm laser and emitted fluorescent light was collected using a 530/30 BP filter (FL-1) or a 695/40 BP filter (FL-3). An overview of fluorochrome specifications, flow cytometry filters and channels used in our experiments is shown in Table S1. The samples were run at a sampling rate between 12.5 and 25 μL/min. The scatter and fluorescence parameters were set to a logarithmic scale. The photomultiplier tube voltage was optimized by using a voltage walk approach to define the optimal separation distances between the unstained and stained populations. The threshold was set on the fluorescence channel FL-1 and on the SSC or FSC to eliminate noise events without excluding particles of interest. This was achieved by successively raising the fluorescence threshold and the SSC or FSC threshold without compromising the size of the detected positive population. The counts of particles per mL measured by flow cytometry were repeated at least four times with different threshold settings and the results were aligned to DPCs measured by microscopy, as described above. In addition, the threshold levels and trigger channels were also determined by acquiring a clean-filtered PBS sample (with or without addition of the dye used for staining of the samples), thereby allowing an event rate of ≤100 events/s when fluorescence threshold triggering was applied or ≤300 events/s when a SSC-based trigger was applied. All samples were diluted in filtered PBS (using a 0.22 µm syringe filter). The measurements were recorded after the fluid stream had stabilized, and the event rate had reached a plateau and had been stable for at least 20 s. To avoid swarm effects, the event rate never exceeded 2500 events/s. The thresholds were adapted in correspondence to the filter and gain settings applied. After finalization of the optimized settings, a workspace was loaded at the beginning of each measurement to calibrate the flow cytometer and to ensure that the measurements were comparable between experiments.

### 5.6. Implementation of the Small Particle Filter

For a better separation of small particles from the background noise, a small particle filter was applied. The difference compared to the standard SSC filter (SSC 488-10+OD2) is the absence of the regularly included neutral density filter with an optical density (OD) of 2. Here, the OD describes the amount of energy that is blocked by the filter. A higher transmission is achieved evenly across a specific spectrum without the neutral density filter. This so-called small particle SSC filter (SSC 488-10) allows even unstained bacteria to be separated from background noise by applying an SSC-based trigger only. Thereby, it was possible to adapt the threshold settings by omitting the fluorescence trigger. This furthermore makes it possible to display the unstained population within the threshold range and enables the usage of additional fluorescence markers at an appropriate event rate of 1000 to 2500 events/s.

### 5.7. Data Acquisition by Flow Cytometry and Statistical Analysis

The resolution of populations for *C. trachomatis* EBs/RBs detected in the SSC and separation from background noise was superior to the resolution in the FSC, especially after implementation of the small particle filter. Therefore, the fluorescence intensity was preferably plotted against the SSC. Furthermore, the most accurate parameter for analyzing small particles is the intensity of the signal displayed as height. For very small particles (with a size smaller than any of the wavelengths of the incident light) [51], the time-of-flight measurements or width become less accurate and consequently also the area, which is the integrated value of the height and width of an electronic pulse. Therefore, the flow cytometry data were displayed in height for all figures except in Figure S1, which shows height (SSC-H) vs. area (SSC-A) plots. The flow cytometry data were analyzed using Flowjo V10.7.1 (FLOWJO, LLC). Prism V7 (GraphPad Software) was used for the statistical analysis and generation of graphs. ImageJ was used for image processing and analysis. Statistical significance was determined between the groups with a one-way analysis of variance (ANOVA) followed by Dunnett’s multiple comparisons test. Significance was set at a *p* value of less than 0.05.

## Figures and Tables

**Table 1:** Comparative measurements of *C. trachomatis* stocks using different methods. Measurements of DPCs of freshly thawed stocks were performed by microscopy and flow cytometry with optimized threshold settings. Counting was performed at the dilution of 1:10,000 of the original stock. The calculated infectivity is the quotient between IFUs and the DPC measured by epifluorescence microscopy.

Stock	Serovar	Method	Concentration of Stock (/mL)		Infectivity (%)	CFDA Staining, Flow Cytometry (%)	SGI/PI Staining, Flow Cytometry (%)
			Mean	SD			
1CTE	E	IFU	2.98 × 10^9^	2.78 × 10^9^			
		DPC-microscopy	2.39 × 10^10^	9.77 × 10^9^	15.4 ± 5.7	38.0 ± 12.5	27.0 ± 3.0
		DPC-flow-cytometry	2.18 × 10^10^	3.90 × 10^9^			
2CTE	E	IFU	6.10 × 10^9^	1.66 × 10^9^			
		DPC-microscopy	2.10 × 10^10^	2.95 × 10^9^	29.5 ± 4.4	60.5 ± 23.8	41.9 ± 1.8
		DPC-flow-cytometry	1.27 × 10^10^	1.07 × 10^5^			
1CTL2	L2	IFU	1.05 × 10^9^	4.96 × 10^7^			
		DPC-microscopy	9.81 × 10^9^	7.26 × 10^8^	10.5 ± 0.8	50.1 ± 2.9	37.8 ± 2.2
		DPC-flow-cytometry	7.60 × 10^9^	7.33 × 10^8^			
1CTF	F	IFU	1.60 × 10^9^	2.37 × 10^8^			
		DPC-microscopy	2.11 × 10^10^	8.79 × 10^9^	8.7 ± 3.3	49.7 ± 3.4	51.8 ± 0.4
		DPC-flow-cytometry	1.88 × 10^10^	1.78 × 10^9^			

## Data Availability

The data presented in this study are available on request from the corresponding author.

## Supplementary Materials

The following are available online at https://www.mdpi.com/article/10.3390/pathogens10121617/s1, Table S1: Overview of fluorochrome specifications and flow cytometry filters/channels used in our experiments. Figure S1: Coincident detection of EBs/RBs (serovar E) stained with SGI, measured at different concentrations with consideration of different thresholds and flow rates. Figure S2: Assessment of bacterial infectivity and viability by flow cytometry. Figure S3: Distinct flow cytometry profiles of different serovars. Figure S4: Flow cytometry profiles of SGI-stained *C. trachomatis* EBs/RBs of serovar E over time.
