# Supplementary material for: A Novel Flow Cytometric Approach for the Quantification and Quality Control of Chlamydia trachomatis Preparations
Source: Pathogens. 2021 Dec 12;10(12):1617. doi: 10.3390/pathogens10121617 (PMC8706156; doi:10.3390/pathogens10121617)
Supplement: Supplementary file 1 [file pathogens-10-01617-s001.zip › pathogens-1493369-SM.pdf]

pathogens

MDPI

Article

A Novel Flow Cytometric Approach for the Quantification and Quality Control of *Chlamydia trachomatis* Preparations

Table S1. Overview of fluorochrome specifications and flow cytometry filters/channels used in our experiments. SGI - SYBR® Green I, CFDA - 5(6) – carboxyfluoresceindiacetate, FITC - fluoresceinisothiocyanat, PI - propidium iodide.

Fluorochrome	Excitation laser (nm)	Exitation$_{max}$(nm)	Emission$_{max}$(nm)	Emission filter (nm)	Channel
SGI	488	494	521	530/30	FL-1
FITC	488	495	520	530/30	FL-1
CFDA	488	495	519	530/30	FL-1
PI	488	535	615	695/40	FL-3

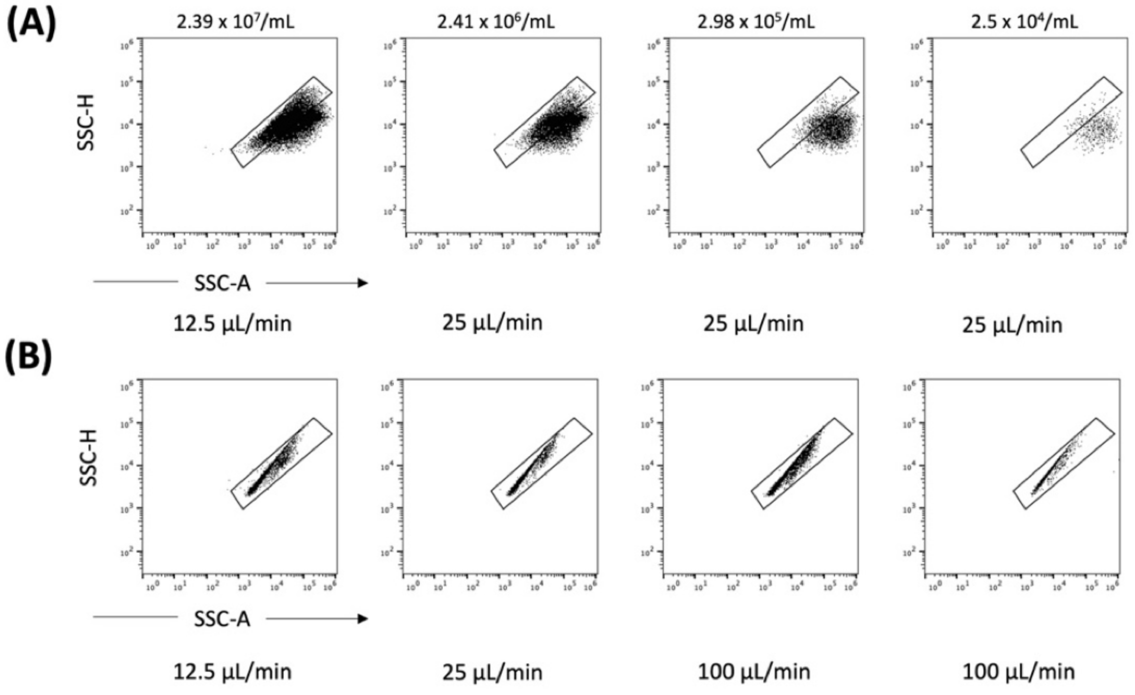

Figure S1. Coincident detection of EBs/RBs (serovar E) stained with SGI, measured at different concentrations with consideration of different thresholds and flow rates. Dot plots (SSC-H vs. SSC-A) of an EB/RB preparation show the coincidence rate measured with the following SSC triggers: **(A)** SSC 100, **(B)** SSC 1,000 and BL-1 1,000, with subsequently increased flow rates, as indicated under the plots.

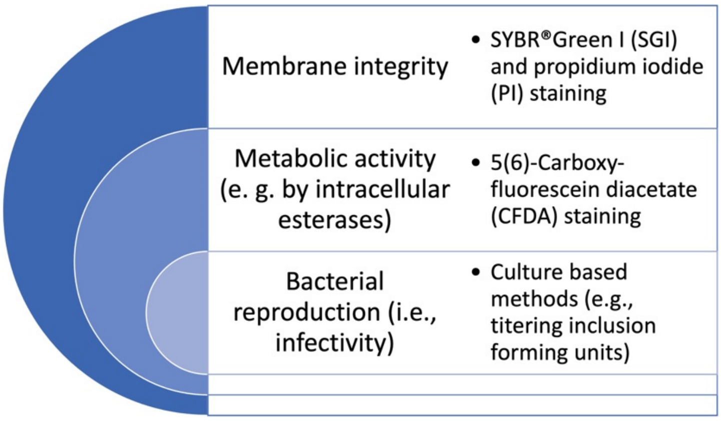

Figure S2. Assessment of bacterial infectivity and viability by flow cytometry. Overview of methods used in this study to detect and count *C. trachomatis* EBs/RBs.

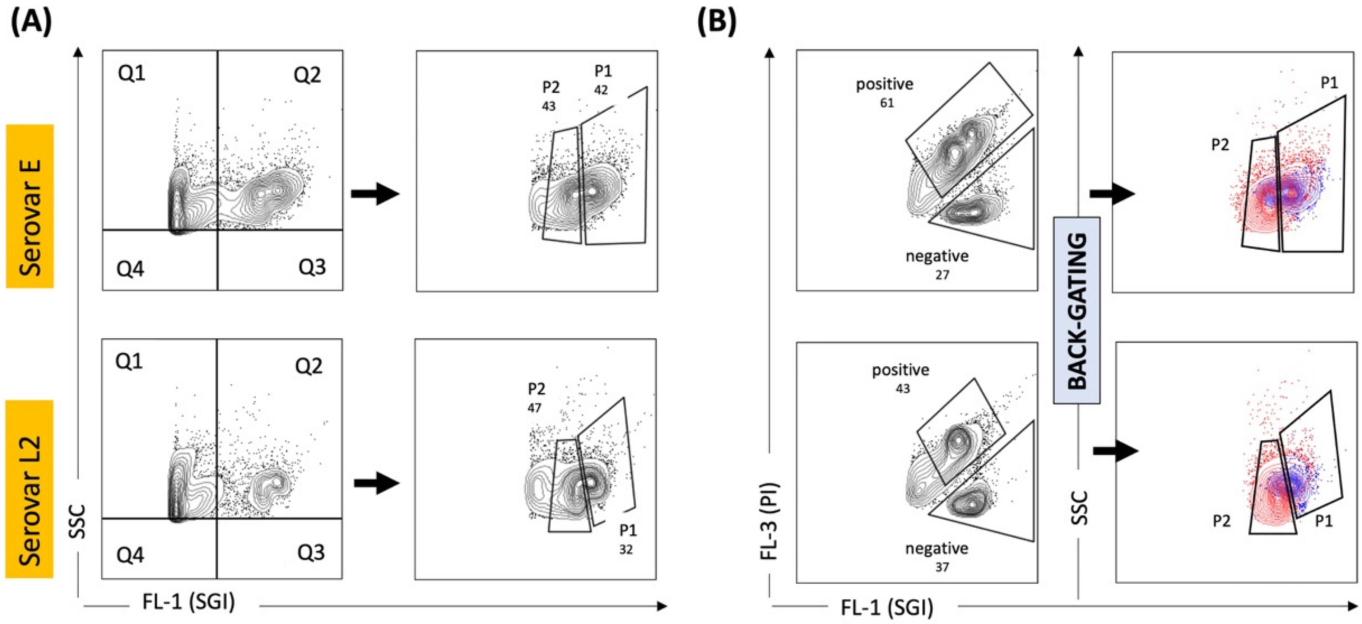

Figure S3. Distinct flow cytometry profiles of different serovars. **(A)** Gating strategies of representative contour plots of SGI/PI-stained EBs/RBs show different flow cytometry profiles of serovar E (upper panel) and serovar L2 (lower panel) separated in the fluorescence channel. The separation of two different populations (P1 and P2) in gate Q2 is shown, the numbers represent percentages. Threshold settings: SSC 1,000 **(B)** The SGI-positive population of both serovars was comparatively analyzed for PI positivity. After back-gating, the PI-positive (red) and the PI-negative (blue) -population is re-located and identified in one of the two positive populations P1 or P2 in the SSC vs. FL-1 (SGI) plot.

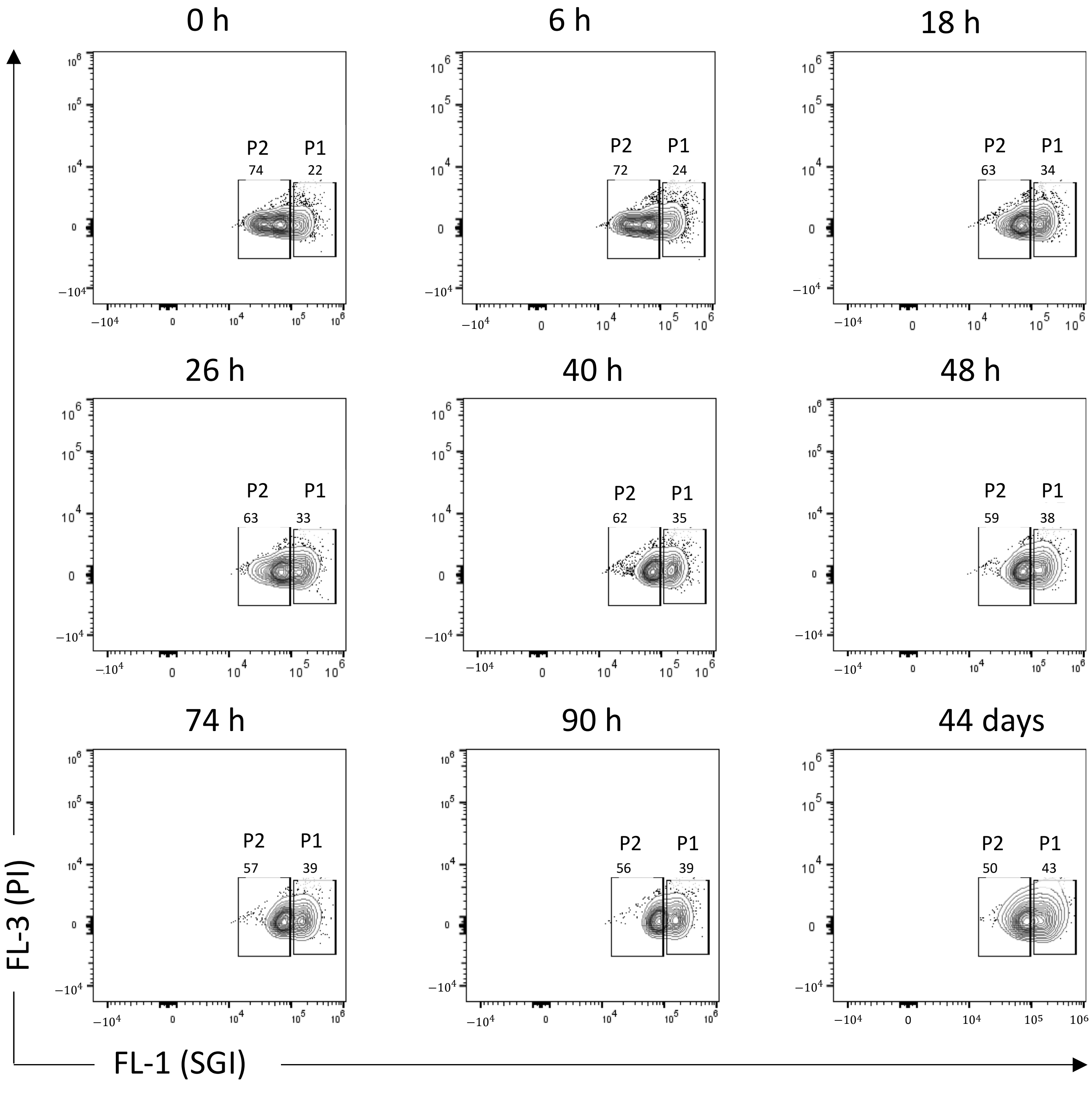

Figure S4. Flow cytometry profiles of SGI-stained *C. trachomatis* EBs/RBs of serovar E over time. Different populations (P1 and P2) in the PI negative gate can be distinguished and alter in a time dependent manner. The numbers represent percentages of bacteria in P1 and P2.
